# Prostaglandin D_2_ metabolites activate asthmatic patient-derived type 2 innate lymphoid cells and eosinophils via the DP_2_ receptor

**DOI:** 10.1186/s12931-021-01852-3

**Published:** 2021-10-07

**Authors:** Saskia Carstensen, Christina Gress, Veit J. Erpenbeck, Shamsah D. Kazani, Jens M. Hohlfeld, David A. Sandham, Meike Müller

**Affiliations:** 1 Department of Biomarker Analysis and Development, Clinical Airway Research, Fraunhofer Institute of Toxicology and Experimental Medicine, Hannover, Germany; 2 Novartis Pharma AG, Basel, Switzerland; 3 Novartis Institutes for Biomedical Research, Cambridge, MA USA; 4 German Center for Lung Research (BREATH), Hannover, Germany; 5 Department of Respiratory Medicine, Hannover Medical School, Hannover, Germany

**Keywords:** Eosinophil shape change, Type 2 innate lymphocyte cells, Asthma, PGD_2_, DP_2_, CRTH2, Fevipiprant, 13,14-Dihydro-15-keto-PGD_2_, PGD_2_ metabolites

## Abstract

**Background:**

Prostaglandin D_2_ (PGD_2_) signaling via prostaglandin D_2_ receptor 2 (DP_2_) contributes to atopic and non-atopic asthma. Inhibiting DP_2_ has shown therapeutic benefit in certain subsets of asthma patients, improving eosinophilic airway inflammation. PGD_2_ metabolites prolong the inflammatory response in asthmatic patients via DP_2_ signaling. The role of PGD_2_ metabolites on eosinophil and ILC2 activity is not fully understood.

**Methods:**

Eosinophils and ILC2s were isolated from peripheral blood of atopic asthmatic patients. Eosinophil shape change, ILC2 migration and IL-5/IL-13 cytokine secretion were measured after stimulation with seven PGD_2_ metabolites in presence or absence of the selective DP_2_ antagonist fevipiprant.

**Results:**

Selected metabolites induced eosinophil shape change with similar nanomolar potencies except for 9α,11β-PGF_2_. Maximal values in forward scatter of eosinophils were comparable between metabolites. ILC2s migrated dose-dependently in the presence of selected metabolites except for 9α,11β-PGF_2_ with EC_50_ values ranging from 17.4 to 91.7 nM. Compared to PGD_2_, the absolute cell migration was enhanced in the presence of Δ^12^-PGD_2_, 15-deoxy-Δ^12,14^-PGD_2_, PGJ_2_, Δ^12^-PGJ_2_ and 15-deoxy-Δ^12,14^-PGJ_2_. ILC2 cytokine production was dose dependent as well but with an average sixfold reduced potency compared to cell migration (IL-5 range 108.1 to 526.9 nM, IL-13 range: 125.2 to 788.3 nM). Compared to PGD_2_, the absolute cytokine secretion was reduced in the presence of most metabolites. Fevipiprant dose-dependently inhibited eosinophil shape change, ILC2 migration and ILC2 cytokine secretion with (sub)-nanomolar potencies.

**Conclusion:**

Prostaglandin D_2_ metabolites initiate ILC2 migration and IL-5 and IL-13 cytokine secretion in a DP_2_ dependent manner. Our data indicate that metabolites may be important for in vivo eosinophil activation and ILC2 migration and to a lesser extent for ILC2 cytokine secretion.

**Supplementary Information:**

The online version contains supplementary material available at 10.1186/s12931-021-01852-3.

## Background

Allergic and non-allergic asthma are closely linked to increased production of prostaglandin D_2_ (PGD_2_) [1–3]. The main source of PGD_2_ are mast cells, which thereby orchestrate early and late immune responses of the innate and adaptive immune system. PGD_2_ primarily signals through prostaglandin D_2_ receptors 1 (DP_1_) and 2 (DP_2_, also known as chemoattractant receptor-homologous molecule expressed on Th2 cells [CRTH2]). Activation of DP_1_ is associated with increased smooth muscle relaxation, vasodilation, vascular permeability as well as reduced leukocyte chemotaxis and cytokine secretion [4, 5]. In contrast, DP_2_ signaling leads to activation, chemokinesis, migration, and cytokine production of various leukocytes such as eosinophils, basophils, T cells or type 2 innate lymphoid cells (ILC2s) [6–11].

**Fig. 1:**
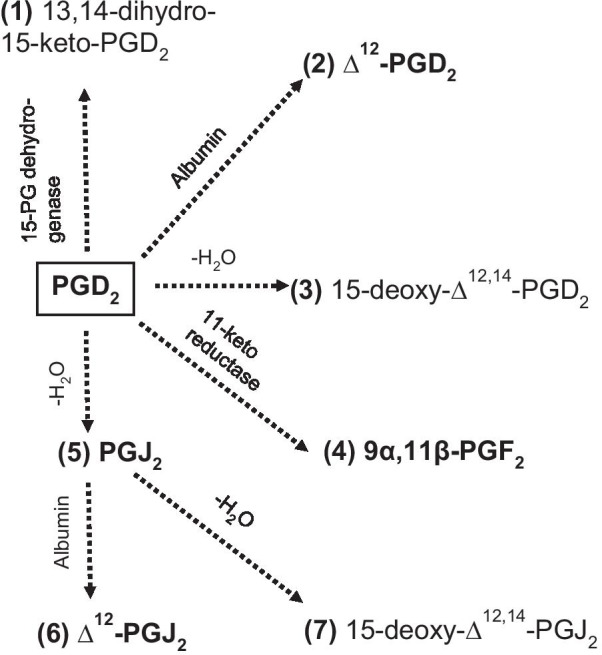
Selected metabolism of prostaglandin D_2_. Major metabolites are represented in bold. PGD_2_, metabolite 2 and 3 belong to the D-series while metabolites 5, 6 and 7 belong to the J-series of prostaglandins. Metabolite 4 belongs to the F-series of prostaglandins

In vivo and in vitro, PGD_2_ is rapidly degraded either enzymatically or spontaneously to various PGD_2_ metabolites of the D, F and J series (Fig. 1) [12, 13]. The persistence of degradation products in plasma and urine may indicate a biological relevance either by preventing or prolonging in vivo receptor signaling [6, 14–16]. Enzymatic degradation of PGD_2_ leads to 13,14-dihydro-15-keto-PGD_2_ (DK-PGD_2_) which is a highly selective DP_2_ agonist [14, 15]. In plasma, PGD_2_ is mainly metabolized to ∆^12^-PGD_2_ and ∆^12^-PGJ_2_ which preferably bind to DP_2_ [12, 15]. Dehydration of PGD_2_ and its spontaneous degradation product 9-deoxy-PGD_2_ (PGJ_2_) lead to formation of 15-deoxy-∆^12,14^-PGD_2_^12,17^ and 15-deoxy-∆^12,14^-PGJ_2_ [7, 17, 18], respectively, which accumulate in low concentrations and as well preferably bind to DP_2_ [15]. A major enzymatically derived product of the PGD_2_ metabolism in vivo is 9α,11β-PGF_2_ which is e.g. found in urine and plasma of asthmatics following allergen challenge [14, 19, 20]. Since most metabolites have selective agonistic properties for DP_2_ over DP_1_ they are considered to contribute to inflammatory DP_2_ signaling [15, 21].

Targeting the DP_2_ pathway is of significant interest for severe, uncontrolled asthmatic patients who do not or only partly respond to current available treatment options [1, 11, 22, 23]. Eosinophils and ILC2s are two major players in atopic asthma expressing DP_2_. PGD_2_ and metabolites activate eosinophils changing their cellular shapes [15, 18, 19], as well as inducing actin polymerization [21], CD11b expression [21] and migration [6, 12]. ILC2 numbers are increased in the airways of asthmatics and promote eosinophilia by secretion of type 2 cytokines [24, 25]. PGD_2_ induces interleukin 5 (IL-5) and IL-13 secretion, cell migration, cell aggregation as well as the expression of adhesion molecules in ILC2s in a DP_2_ dependent manner [26, 27]. Further, ILC2s produce endogenous PGD_2_ which is degraded to PGJ_2_, ∆^12^-PGJ_2_, 15-deoxy-∆^12,14^-PGJ_2_ and DK-PGD_2_ in culture supernatants [28].

Fevipiprant is a potent and selective DP_2_ antagonist that has shown therapeutic benefit in certain subsets of asthma patients in phase 2 clinical trials [29–31]. In a single center mechanistic phase 2 clinical trial in patients with persistent eosinophilic asthma, fevipiprant not only reduced airway inflammation, but also improved epithelial integrity and reduced airway smooth muscle mass [31, 32]. In two recently published phase 3 studies in severe asthmatics, although neither trial showed a statistically significant reduction in asthma exacerbations, consistent and modest reductions in exacerbations rates were observed in both studies with a high dose of fevipiprant [33]. In two Phase 3 studies in moderate asthmatics, no significant improvements were observed in lung function or other asthma related outcomes, such as daytime symptom score or quality of life [34]. Previously, the inhibitory effects of fevipiprant in primary human cells have been characterized with PGD_2_ activation [26, 35, 36].

Here, we demonstrate the potencies of seven PGD_2_ metabolites to induce ILC2 migration and IL-5 and IL-13 cytokine secretion. We also reproduce previously reported effects of the PGD_2_ metabolites on eosinophil shape change. ILC2 as well as eosinophil activation was then blocked with fevipiprant demonstrating the DP_2_ dependency of cell activation.

## Methods

### Study design

Fifteen atopic asthmatic volunteers (eight male/seven female; aged 18–65 years, average 34.5 ± 10.6 years; BMI 19–32 kg/m^2^, no oral steroids for > four weeks) were enrolled into the study. Subjects differed between eosinophil (n = 8) and ILC2 (n = 7) experiments and also between activation and inhibition experiments, respectively. Whole blood (200–500 ml) was collected in 3.8% trisodium citrate and was processed within one hour after blood withdrawal. Eosinophil levels in peripheral blood had to be > 0.15 × 10^6^/ml for the eosinophil shape change.

### Reagents

PGD_2_, 13,14-dihydro-15-keto-PGD_2_, PGJ_2_, Δ^12^-PGJ_2_, Δ^12^-PGD_2_, 15-deoxy-Δ^12,14^-PGJ_2_, 15-deoxy-Δ^12,14^-PGD_2_, 9α,11β-PGF_2_ were purchased from Cayman Chemicals (Biomol GmBH, Hamburg, Germany). Fevipiprant (GST0000013789) was provided by Novartis Pharma AG (Basel, Switzerland). Reagents were dissolved in sterile-filtered Hybri-Max dimethylsulfoxide (DMSO, Sigma-Aldrich, Taufkirchen, Germany).

### Granulocyte isolation

Blood was stored on ice until processing. Granulocytes were isolated as described [18, 37]. In brief, 200 ml blood was diluted 1:3 in Dulbecco’s Phosphate Buffered Saline (DPBS). The blood suspension was incubated with 4% (w/v) dextran-T500 (dilution 5:1, VWR, Hannover, Germany) for 30 min on ice. The upper phase was layered on Ficoll-Paque® (Sigma-Aldrich, Taufkirchen, Germany) and centrifuged (25 min, 300×*g*, 18 °C). Granulocyte pellets each were resuspended in 500 µl DPBS and erythrocytes were lysed for 40 s with 20 ml ice cold, sterile, endotoxin-free distilled water. The reaction was stopped with 20 ml DPBS (2 × concentrated). After centrifugation (10 min, 300×*g*, 18 °C), cell pellets were washed with 50 ml DPBS. Granulocytes were resuspended in assay buffer (0.1% bovine serum albumin (BSA) in DPBS, Sigma-Aldrich, Taufkirchen, Germany) to a cell density of 6.25 × 10^6^/ml.

### Eosinophil shape change

Granulocytes (80 µl) and assay buffer (10 µl) were incubated in a water bath (5 min, 37 °C). Metabolite solutions (10 µl, final concentration (conc.) 0.01 nM, 0.1 nM, 0.5 nM, 1 nM, 5 nM, 10 nM, 100 nM, 1 µM, 1% DMSO in assay buffer) were added (5 min, 37 °C). The incubation was stopped with 250 µl of 0.25% BD Cell-Fix™ Solution (1:10 with sterile water followed by 1:4 with assay buffer, BD Biosciences, Heidelberg, Germany) and immediate placement on ice (≥ 5 min). Flow cytometric analysis was performed with a Navios 3/10 (Beckman Coulter). Granulocytes were determined by FSC and SSC properties. Eosinophils were discriminated from neutrophils by autofluorescence properties at 560 nm (FL2). 1000 eosinophils were acquired. Eosinophil shape change was calculated as percentage increase of the mean FSC units.

For DP_2_ inhibition experiments, granulocytes (80 µl) and fevipiprant (10 µl, final conc. 0.01 nM, 0.05 nM, 0.1 nM, 1 nM, 5 nM, 10 nM, 100 nM, 500 nM, 1 µM, 10 µM) were incubated in a water bath (5 min, 37 °C). Metabolite solution (10 µl, EC_70_ concentration) was added for 5 min (37 °C). The reaction was stopped and cells were analyzed as described above.

### Type 2 innate lymphoid cell isolation

Blood was stored at RT until processing. Whole blood (500 ml) was diluted 1:2 in DPBS. PBMCs were isolated using Ficoll-Paque® and SepMate-50 PBMC Isolation tubes (STEMCELL Technologies, Grenoble, France) following manufacturer’s protocol. PBMCs were pooled, washed with 50 ml DPBS and centrifuged (5 min, 300×*g*, 4 °C). T cells, B cells and monocytes depletion enriched ILC2s using CD3, CD14 and CD19 MACS separation beads (Miltenyi Biotech, Bergisch Gladbach, Germany) and LD columns following manufacturer’s protocol. Enriched cells were centrifuged (5 min, 300×*g*, 4 °C) and resuspended in staining buffer containing 5% fetal bovine serum (FBS) and 2 mM ethylenediaminetetraacetic acid (EDTA) in DPBS. Cells were stained for 15 min at RT with a PerCP-Cy5.5-labeled lineage cocktail (CD4, CD8, CD14, CD16, CD19, CD34, CD123, FcεRI), CD11b-FITC, CD56-FITC, CD3-BV510, CD127-BV421, CD45-Alexa Fluor 700 and CD294-PE. View supplement for more detailed information. CD45+, Lineage-, CD11b-, CD56-, CD3-, CD127+, CD294+ cells were sorted with an FACS ARIA Fusion (BD Bioscience) into 96 U bottom well plates (Corning, Amsterdam, Netherlands).

### Type 2 innate lymphoid cell culture

Sorted ILC2s (100 cells/well) were expanded with human feeder PBMCs (100,000/well; 37 °C, 5% CO_2_) for three to five weeks. Culture medium contained RPMI 1640 Glutamax medium (Life Technologies, Darmstadt, Germany), 1% Pen/Strep (Life Technologies, Darmstadt, Germany), 10% *h.i.* human AB serum (Sigma-Aldrich, Taufkirchen, Germany), and 25 mM 4-(2-hydroxyethyl)-1-piperazineethanesulfonic acid **(**HEPES, Lonza, Wakersville, USA). The medium was supplemented with 100 U/ml rh-IL-2 (Life Technologies, Darmstadt, Germany), 25 ng/ml rh-IL-4 (Miltenyi Biotech, Bergisch Gladbach, Germany), 5 µg/ml phytohemagglutinin-M (PHA-M; Sigma-Aldrich, Taufkirchen, Germany).

### Cell migration assay

Migration was assessed using 5.0 µm pore size, polycarbonate membrane, polystyrene 96-transwell plates (Corning, Amsterdam, Netherlands; Hölzel, Köln, Germany) following manufacturer’s protocols. Metabolites (lower well, final conc. 5 nM, 10 nM, 50 nM, 100 nM, 500 nM, 1 µM, 2 µM, 3 µM and 5 µM) and cells (upper well, ≤ 100,000/well) were incubated for 6 h (37 °C, 5% CO_2_) in culture medium without IL-2, IL-4 and PHA. Migrated cells (25 µl) were incubated with CellTiter-Glo® Luminescence Cell Viability Assay (Promega, Mannheim, Germany) following manufacturer’s protocol. Luminescence was measured using a Tecan infinite F200 pro.

For DP_2_ inhibition experiments, ILC2s were incubated with fevipiprant (final conc. 0.01 nM, 0.1 nM, 0.5 nM, 1 nM, 5 nM, 10 nM, 100 nM, 1 µM, 10 µM, for 1 h, 37 °C, 5% CO_2_) in culture medium without IL-2, IL-4 and PHA and cell migration was measured as described above using the EC_70_ concentration of respective metabolites.

### Cytokine measurement

Cells (≤ 150,000/well) and metabolite solutions (final conc. 2.5 nM, 5 nM, 25 nM, 50 nM, 250 nM, 500 nM, 1 µM, 1.5 µM, 2.5 µM) were incubated for 24 h in culture medium without IL-2, IL-4 and PHA (U-bottom 96-well plates, 37 °C, 5% CO_2_). After centrifugation (5 min, 300×*g*, RT), the supernatant was collected and stored at − 80 °C until measurement.

For DP_2_ inhibition experiments, ILC2s were incubated with fevipiprant (final conc. 0.01 nM, 0.1 nM, 0.5 nM, 1 nM, 5 nM, 10 nM, 50 nM, 100 nM, 1 µM) for 1 h (37 °C, 5% CO_2_) and afterwards with the EC_70_ concentration of respective metabolites for 24 h. Cells were incubated as described above.

Supernatants were diluted 1:10 and IL-5 and IL-13 concentrations were measured by MSD immunoassays (V-PLEX Meso Dale Discovery, Rockville, MD, USA) following manufacturer’s protocol. Raw values are depicted in Additional file 1: Figs. S2 and S3.

### Statistics

GraphPad Prism 9.0.1 was used for analysis. Four parameter non-linear agonist or inhibitor regression models were used fit curves and to calculate EC_50_/IC_50_ values, respectively. Fitting curves constraints are indicated in respective figure legends.

Maximal PGD_2_-induced responses were compared with respective maximal responses of metabolites using paired One-Way ANOVA with post-hoc Dunn’s multiple comparisons tests.

## Results

### PGD_2_ metabolites induce eosinophil shape change with similar potencies

PGD_2_ and seven PGD_2_ metabolites were evaluated for their ability to induce activation of eosinophils by measurement of cellular shape changes. All prostaglandins induced a concentration dependent shape change with nanomolar potency except for 9α,11β-PGF_2_ which was less potent (Additional file 1: Fig. S1). EC_50_ values of prostaglandins, described as mean ± standard error of the mean (SEM), fell into following rank order: PGD_2_: 0.7 ± 0.2 nM < Δ^12^-PGD_2_: 1.2 ± 1.8 nM < 15-deoxy-Δ^12,14^-PGD_2_: 1.5 ± 1.6 nM < PGJ_2_: 1.6 ± 3.8 nM < DK-PGD_2_: 2.7 ± 2.3 nM < Δ^12^-PGJ_2_: 5.6 ± 1.0 nM < 15-deoxy-Δ^12,14^-PGJ_2_: 12.0 ± 0.7 nM < 9α,11β-PGF_2_: > 1000. Similar EC_50_ values were reported by Sandig et al. but with different rank order (Table 1) [19].

**Table 1:** EC_50_ values of PG-induced eosinophil shape change were calculated and are given as mean ± standard error of the mean (SEM), n = 2–3

Eosinophil shape change EC_50_ [nM]		
Metabolite	Measured values	Sandig et al. [19]
PGD_2_	0.7 ± 0.2	0.4
DK-PGD_2_	2.7 ± 2.3	1.1
∆^12^-PGD_2_	1.2 ± 1.8	7.3
15-deoxy-∆^12,14^-PGD_2_	1.5 ± 1.6	2.4
9α,11β-PGF_2_	> 1000	156.0
PGJ_2_	1.6 ± 3.8	2.2
∆^12^-PGJ_2_	5.6 ± 1.0	3.7
15-deoxy-∆^12,14^-PGJ_2_	12.0 ± 0.7	8.4

Published reference values are given in the right column

**Fig. 2:**
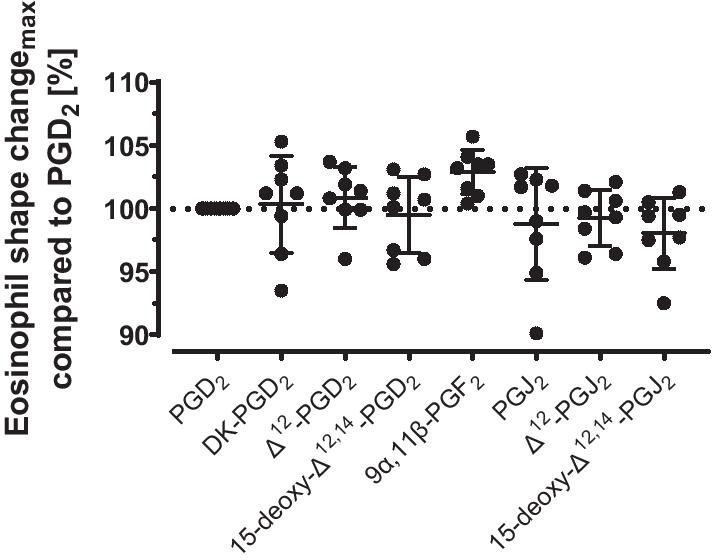
Comparison of maximal eosinophil shape change in the presence of PGD_2_ and selected metabolites. The mean forward scatter values in the presence of the highest PGD_2_ concentration was normalized to 100% shape change, n = 6–8. Values are given as mean ± SD

Metabolites induced shape changes with similar maximal responses as compared to PGD_2_, which was observed by a comparison of FSC_max_ values normalized on 100% PGD_2_ shape change. Activation responses of PGD_2_ metabolites ranged between 98.0 and 102.9%, compared to the parent PGD_2_ (Fig. 2). Sandig et al*.* did not compare maximal responses but their results indicate similar responses for most metabolites as well while responses of Δ^12^-PGD_2_ and Δ^12^-PGJ_2_ seemed to have higher FCS values.

### PGD_2_ metabolites induce ILC2 migration and IL-5 and IL-13 cytokine secretion

**Fig. 3:**
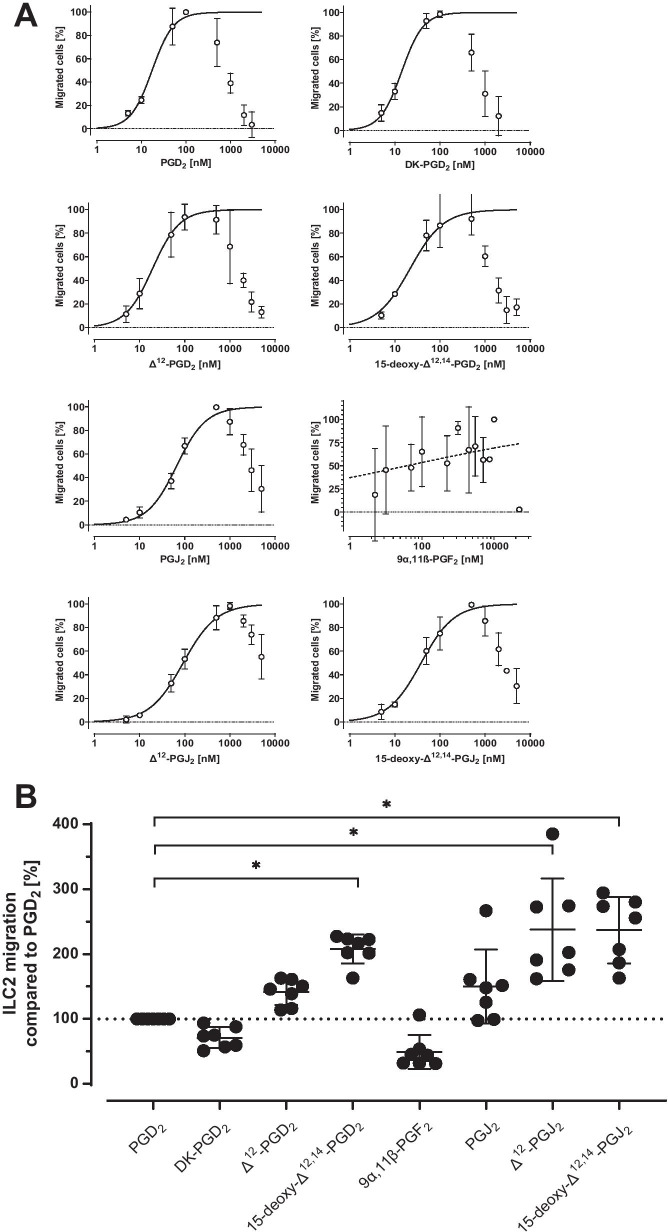
**A** ILC2 migration in the presence of ascending concentrations of PGD_2_ and selected metabolites, n = 3. Values are given as mean ± SD. Nonlinear curve fit with constraints of bottom constant equal to zero and top constant equal to 100 was applied to derive EC_50_ and EC_70_. **B** Maximal cell migration of PGD_2_ and selected metabolites. Absolute numbers of migrated cells were compared and normalized to 100% PGD_2_, n = 7. Values are given as mean ± SD. DK-PGD_2_: ns, ∆^12^-PGD_2_: ns, 15-deoxy-∆^12,14^-PGD_2_: p = 0.05, 9α,11β-PGF_2_: ns, PGJ_2_: ns, ∆^12^-PGJ_2_: p = 0.03 and 15-deoxy-∆^12,14^-PGJ_2_: p = 0.01. *p < 0.05

Next, PGD_2_ metabolites were evaluated for their potential to induce ILC2 migration and Type 2 cytokine secretion. The metabolites induced cell migration concentration dependently and with nanomolar potency except for 9α,11β-PGF_2_. 9α,11β-PGF_2_ increased migration in comparison to the medium control but dose independently (Fig. 3A). Migratory potencies were increased for the D-series of metabolites in comparison to the J-series with mean EC_50_ ± SEM values of PGD_2_ 17.4 ± 3.9 nM, DK-PGD_2_ 14.2 ± 3.4 nM, ∆^12^-PGD_2_ 19.3 ± 3.2 nM, 15-deoxy-∆^12,14^-PGD_2_ 21.8 ± 6.3 nM, PGJ_2_ 66.3 ± 7.0 nM, ∆^12^-PGJ_2_ 91.7 ± 9.2 nM and 15-deoxy-∆^12,14^-PGJ_2_ 38.1 ± 5.4 nM (Table 2). Micromolar metabolite concentrations abolished cell migration.

**Table 2:** EC_50_ values of PG-induced ILC2 migration and IL-5 and IL-13 cytokine secretion were calculated and are given as mean ± standard error of the mean (SEM), n = 3

ILC2 migration and cytokine secretion EC_50_ [nM]			
Metabolite	Migration	IL-5 secretion	IL-13 secretion
PGD_2_	17.4 ± 3.9	139.2 ± 15.5	131.9 ± 10.8
DK-PGD_2_	14.2 ± 3.4	146.0 ± 18.0	159.2 ± 28.8
∆^12^-PGD_2_	19.3 ± 3.2	108.1 ± 11.8	125.2 ± 8.3
15-deoxy-∆^12,14^-PGD_2_	21.8 ± 6.3	175.4 ± 26.4	159.1 ± 13.9
9α,11β-PGF_2_	–^a^	526.9 ± 12,926.7	788.3 ± 173.7
PGJ_2_	66.3 ± 7.0	244.4 ± 26.3	321.9 ± 43.8
∆^12^-PGJ_2_	91.7 ± 9.2	234.1 ± 30.6	343.2 ± 52.4
15-deoxy-∆^12,14^-PGJ_2_	38.1 ± 5.4	185.3 ± 21.5	226.2 ± 35.2

^a^Curve fit was ambiguous, EC_50_ value could not be determined

The maximal migration response measured by total number of migrated cells compared to PGD_2_ was increased for ∆^12^-PGD_2_, 15-deoxy-∆^12,14^-PGD_2_, PGJ_2_, ∆^12^-PGJ_2_ and 15-deoxy-∆^12,14^-PGJ_2_. In contrast DK-PGD_2_ and 9α,11β-PGF_2_ showed a non-significant trend to reduced maximal migration responses, (Fig. 3B).

**Fig. 4:**
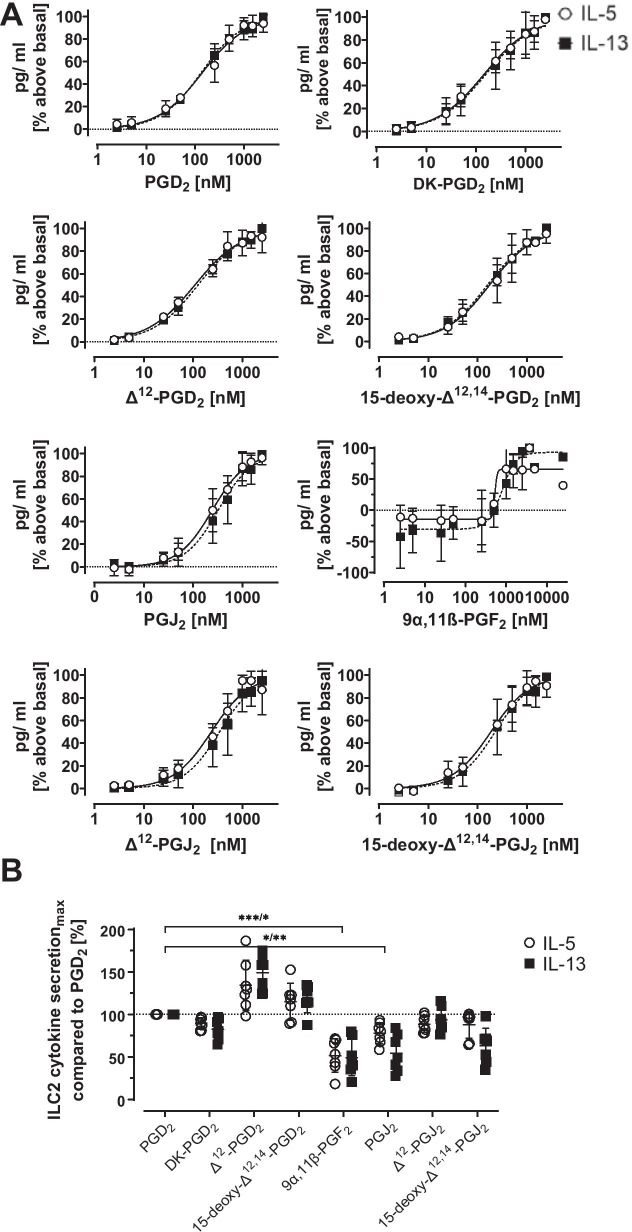
**A** IL-5 (white circles) and IL-13 (black squares) cytokine secretion of ILC2s in the presence of ascending concentrations of PGD_2_ and selected metabolites, n = 3. Values are given as mean ± SD. Nonlinear curve fit with constraints of bottom constant equal to zero and top constant equal to 100 was applied to derive EC_50_ and EC_70_. **B** Maximal cytokine secretion of selected metabolites compared to PGD_2_. The absolute concentrations of secreted cytokines were compared and normalized to 100% PGD_2_, n = 6. Values are given as mean ± SD. 9α,11β-PGF_2_: IL-5 p = 0.006, IL-13 p = 0.02, PGJ_2_: IL-5 p = 0.03, IL-13 p = 0.007. *p < 0.05, **p < 0.01, ***p < 0.001, ****p < 0.0001

IL-5 and IL-13 cytokine secretion of ILC2s was induced by PGD_2_ metabolites in a concentration dependent manner. ILC2 responses were comparable between IL-5 and IL-13 (Fig. 4A). Similar to cell migration, higher potencies for cytokine secretion were found for the D-series of metabolites compared to the J-series (Table 2). 9α,11β-PGF_2_ was able to induce cytokine secretion although less potent than the other metabolites and with high variability. The maximal cytokine secretion response was significantly lower in the presence of 9α,11β-PGF_2_ and PGJ_2_ compared to PGD_2_ while the maximal response was similar for DK-PGD_2_, ∆^12^-PGJ_2_ and 15-deoxy-∆^12,14^-PGJ_2_. Cytokine secretion was non-significantly enhanced for ∆^12^-PGD_2_ and 15-deoxy-∆^12,14^-PGD_2_ compared to PGD_2_ (Fig. 4B).

### Selective DP_2_ inhibition of eosinophils and ILC2s

**Fig. 5:**
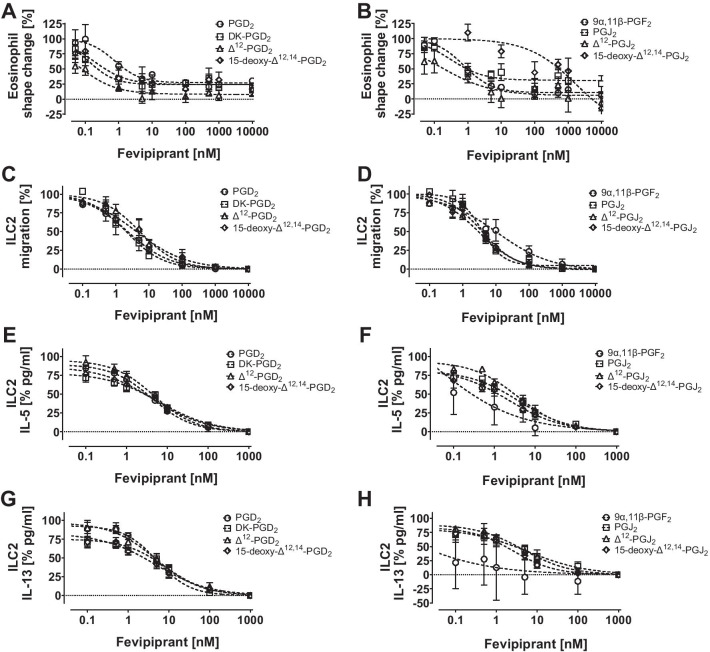
Inhibition of PGD_2_ and metabolite-induced cell activation with DP_2_ inhibitor fevipiprant. Cells were incubated with ascending concentrations of fevipiprant and stimulated with EC_70_ concentrations of PGD_2_, DK-PGD_2_, ∆^12^-PGD_2_, 15-deoxy-∆^12,14^-PGD_2_, PGJ_2_, 9a,11b-PGF_2_, ∆^12^-PGJ_2_ and 15-deoxy-∆^12,14^-PGJ_2_, respectively. **A**, **B** Inhibition of eosinophil shape change, n = 3–5. Top constraints equal 100. **C**, **D** Inhibition of ILC2 migration, n = 4. Top constraints equal 100. **E**, **F** Inhibition of ILC2 IL-5 secretion, n = 4. Bottom constraints equal zero. (G,H) Inhibition of ILC2 IL-13 secretion, n = 4. Bottom constraints equal zero. Values are given as mean ± SEM

Isolated whole blood eosinophils were incubated with the selective DP_2_ antagonist fevipiprant prior to activation of cells with EC_70_ concentrations of PGD_2_ or respective metabolites (supplementary table 2). Fevipiprant inhibited eosinophil shape changes in dose dependent manners with sub-nanomolar potencies (Fig. 5A and B). IC_50_ values against metabolites were comparable and ranged between 0.1 and 0.9 nM for PGD_2_ and six metabolites (Table 3). Eosinophil shape changes induced by 15-deoxy-∆^12,14^-PGJ_2_ could be inhibited by fevipiprant, however, the dose–response was not sigmoidal.

**Table 3:** IC_50_ values of fevipiprant-inhibited eosinophil shape change, ILC2 migration and IL-5 and IL-13 cytokine secretion of ILC2s

IC_50_ fevipiprant [nM]				
Metabolite	shape change (n = 3–5)	Migration (n = 4)	IL-5 (n = 4)	IL-13 (n = 4)
PGD_2_	0.9 ± 0.2	2.4 ± 0.6	4.2 ± 0.8	5.1 ± 1.1
DK-PGD_2_	0.1 ± 0.0	1.9 ± 0.7	6.5 ± 1.6	8.5 ± 2.1
∆^12^-PGD_2_	0.1 ± 0.0	4.9 ± 1.3	4.8 ± 1.2	4.8 ± 1.3
15-deoxy-∆^12,14^-PGD_2_	0.5 ± 0.3	4.6 ± 2.0	4.5 ± 1.7	4.6 ± 1.4
9α,11β-PGF_2_	0.9 ± 0.1	10.9 ± 7.8	–^a^	–^a^
PGJ_2_	0.5 ± 0.2	3.7 ± 0.6	4.0 ± 1.0	5.9 ± 3.6
∆^12^-PGJ_2_	0.6 ± 0.3	3.1 ± 0.5	3.7 ± 0.9	4.6 ± 1.6
15-deoxy-∆^12,14^-PGJ_2_	–^a^	3.8 ± 0.8	2.3 ± 0.9	2.6 ± 0.9

Values were calculated from 3 to 5 experiments and are given as mean ± standard error of the mean (SEM)

^a^Curve fit was ambiguous, IC_50_ values could not be determined

Similar to eosinophil activation, ILC2 migration (Fig. 5C and D) and cytokine secretion (Fig. 5E–H) could be inhibited by fevipiprant in a concentration dependent manner. In comparison to eosinophils, ILC2 related IC_50_ values were approximately one order of magnitude higher. Migration IC_50_ values ranged between 1.9 and 10.9 nM, IL-5 IC_50_ values ranged between 2.3 and 6.5 nM, and IL-13 IC_50_ values ranged between 2.6 and 8.5 nM (Table 3). 9a,11b-PGF_2_ induced cytokine secretion results were highly variable among donors and IC_50_ values could not be determined.

## Discussion

In vivo and in vitro, PGD_2_ is rapidly degraded to various metabolites which presumably contribute to DP_2_ immune cell activation in large part due to their DP_2_ selectivity [15, 38]. Here, we show for the first time that ILC2s respond to PGD_2_ metabolites by migration and IL-5 and IL-13 secretion. We determined respective EC_50_ values, quantified cellular responses of eosinophils and ILC2s and compared the activation maximal responses with respective PGD_2_ responses. DP_2_ dependency was demonstrated using fevipiprant which abolished cell activities with nanomolar potencies.

The influence of prostaglandin metabolites on eosinophil cell activation and shape change is well known. Sandig et al*.* first reported EC_50_ values, which we could largely confirm [19]. EC_50_ concentrations resulted in different rank orders of potencies compared to ours, however, those differences were negligibly small. The potency of 9α,11β-PGF_2_ was clearly reduced in both studies compared to other metabolites. Since 9α,11β-PGF_2_ has an approximately 130 fold reduced binding affinity to DP_2_ compared to PGD_2_ [38], it can be assumed that the concentrations used were too low to sufficiently induce a shape changes in eosinophils. Merely concentrations of 1 µM demonstrated a shift in FCS properties (Additional file 1: Fig. S1). Comparing the maximal changes in the forward scatters indicated that all metabolites induced shape changes with similar maximal responses. This may imply their comparable agonistic function on eosinophil activation. This finding could be supported by further eosinophil activation data addressing e.g. cell surface CD11b expression or degranulation.

The selective relevance of DP_2_ for eosinophil activation has been reported for PGD_2_ and DK-PGD_2_ using various DP_2_ antagonists [19, 39, 40]. Nevertheless, blocking the activation of other metabolites was described only using the dual DP_2_ and thromboxane receptor (TP) antagonist ramatroban [19]. We complemented data affirming that metabolites induced eosinophil shape changes via DP_2_ selectively using fevipiprant [41].

Less is known about the role of PGD_2_ metabolites on ILC2 activation. Recently it was shown that ILC2s produce endogenous PGD_2_ upon alarmin activation which was metabolized to PGJ_2_, ∆^12^-PGJ_2,_ DK-PGD_2_ and 15-deoxy-∆^12,14^-PGJ_2_ in culture supernatants [28]. However, so far only exogeneous and endogeneous PGD_2_ was reported to induce migration and IL-5 and IL-13 secretion in ILC2s [26–28]. Here, we show that ILC2s also migrate and release Type 2 cytokines in presence of DK-PGD_2_, PGJ_2_, Δ^12^-PGJ_2_, Δ^12^-PGD_2_, 15-deoxy-Δ^12,14^-PGJ_2_, and 15-deoxy-Δ^12,14^-PGD_2_. D-series metabolites were more potent than those of the J-series. 9α,11β-PGF_2_ was not able to induce ILC2 migration in the concentration range used. The comparably low binding affinity of 9α,11β-PGF_2_ to DP_2_ might have resulted in the low response. Varying activating potencies among the other metabolites might be related to differences in respective DP_2_ binding affinities as well [15, 38], however, EC_50_ rank orders of shape change, migration and cytokine release experiments were different. Moreover, quantifying the maximal assay responses among metabolites revealed that ILC2 cell migration was enhanced in presence of most metabolites compared to PGD_2_ while cytokine production was not enhanced. Interestingly, cell migration was strongest for the J-series metabolites Δ^12^-PGJ_2_, 15-deoxy-Δ^12,14^-PGJ_2_ and cytokine secretion was strongest for the D-series metabolites Δ^12^-PGD_2_, 15-deoxy-Δ^12,14^-PGD_2_. Our data may indicate that PGD_2_ metabolites contribute predominantly to ILC2 migration rather than cytokine secretion which needs to be confirmed in vivo. Although it is known that numbers of eosinophils and ILC2s are elevated in the airways of severe asthmatic patients [24] little is known about local distributions of PGD_2_ metabolite concentrations in human tissues. ILC2 numbers and PGD_2_ concentrations in the bronchoalveolar lavage (BAL) fluid were reported to correlate after allergen challenge of mild asthmatics [25]. Moreover, metabolic products of PGD_2_ were found in human serum [42], plasma, BAL [43] and urine [44, 45] samples, however, their contribution to the asthmatic disease is poorly understood [20]. Studies on eosinophils in mice showed that ∆^12^-PGJ_2_ mobilizes eosinophils from the bone marrow and facilitates cell extravasation [18] which fits with the observation that PGD_2_ is preferably converted to ∆^12^-PGD_2_ and ∆^12^-PGJ_2_ in blood plasma [12]. The contribution of metabolites to cell recruitment might be of biological relevance but remains speculative without further experiments. 9α,11β-PGF_2_ is another major degradation products found in human and showed low agonistic potency on eosinophils and ILC2s [42, 44, 46]. At micromolar concentrations, 9α,11β-PGF_2_ was able to activate cells, which probably do not represent physiological concentrations of the metabolite. Further, quantification of ILC2 responses indicated a reduced maximal response compared to PGD_2_. Therefore, it seems more likely that 9α,11β-PGF_2_ does not contribute to DP_2_ signaling in vivo but rather serves as an signal-inactivating degradation product.

Blocking with fevipiprant confirmed the DP_2_ dependency of ILC2 activities which were comparable to previous experiments performed with PGD_2_ [26]. Inhibition of 15-deoxy-Δ^12,14^-PGJ_2_ activation in eosinophils followed a non-sigmoidal dose–response principle which should be assessed more closely in future studies. Inhibiting PGD_2_ signaling has been studied in a wide range of clinical studies due to its key role in allergic inflammation. While fevipiprant itself is no longer being developed for asthma, our findings may be of relevance to other DP_2_ antagonists which remain under clinical investigation [22, 47].

## Conclusion

PGD_2_ metabolites are effective DP_2_ agonists and promote eosinophil shape change, ILC2 cell migration and Type 2 cytokine secretion in vitro. They might contribute to ILC2 recruitment in vivo since predominantly ILC2 migration but not ILC2 cytokine secretion or eosinophil shape change was enhanced compared to PGD_2_.

## Supplementary Information

**Additional file 1: Table S1**. Detailed information on flow cytometric antibodies used for cell sorting of ILC2s. **Table S2.** Calculated agonist EC70 values for PGD2 and seven selected PGD2 metabolites. **Table S3.** Characteristics of study subjects. All subjects had a history of allergic asthma since at least 12 months. BMI had to be between 19 to 32 kg/ m2. **Figure S1.** Eosinophil shape change induced by PGD2, DK-PGD2, ∆12-PGD2, 15-deoxy-∆12,14-PGD2, PGJ2, 9a,11b-PGF2, ∆12-PGJ2 and 15-deoxy-∆12,14-PGJ2. Granulocytes were isolated from whole blood of asthmatic patients and were incubated with increasing concentrations of metabolites, n=3, 9α,11β-PGF2: n=2. The mean fluorescence values of the forward scatter were determined by flow cytometry and the percentage of shape change above basal was calculated. Values are given as mean ± SD. **Figure S2.** Concentration of IL-5 cytokine secretion of ILC2s in the presence of ascending concentrations of PGD2 and selected metabolites, n=3. Values are given for three different subjects (circle, square and triangle). **Figure S3.** Concentration of IL-13 cytokine secretion of ILC2s in the presence of ascending concentrations of PGD2 and selected metabolites, n=3. Values are given for three different subjects (circle, square and triangle).

## Data Availability

The datasets supporting the conclusion of this article are available on reasonable request from MM and DAS.

## Abbreviations

BAL: Bronchoalveolar lavage
BMI: Body mass index
BSA: Bovine serum albumin
CD: Cluster of differentiation
Conc.: Concentration
CRTH2: Chemoattractant receptor-homologous molecule expressed on Th2 cells
DK-PGD_2_: 13,14-Dihydro-15-keto-prostaglandin D_2_
DMSO: Dimethylsulfoxide
DP: D-prostanoid receptor
DPBS: Dulbecco’s Phosphate Buffered Saline
EC_50_: Half maximal effective concentration
EDTA: Ethylenediaminetetraacetic acid
FSC: Forward scatter
HEPES: 4-(2-Hydroxyethyl)-1-piperazineethanesulfonic acid
IC_50_: Half maximal inhibitory concentration
IL: Interleukin
ILC2: Type 2 innate lymphoid cells
MFI: Mean fluorescence intensity
PG: Prostaglandin
PHA-M: Phytohemagglutinin-M
SD: Standard deviation
SEM: Standard error of mean
SSC: Sideward scatter
