# Supplementary material for: Prostaglandin D2 metabolites activate asthmatic patient-derived type 2 innate lymphoid cells and eosinophils via the DP2 receptor
Source: Respir Res. 2021 Oct 7;22:262. doi: 10.1186/s12931-021-01852-3 (PMC8499518; doi:10.1186/s12931-021-01852-3)
Supplement: Supplementary file 1 — Additional file 1: Table S1. Detailed information on flow cytometric antibodies used for cell sorting of ILC2s. Table S2. Calculated agonist EC70 values for PGD2 and seven selected PGD2 metabolites. Table S3. Characteristics of study subjects. All subjects had a history of allergic asthma since at least 12 months. BMI had to be between 19 to 32 kg/ m2. Figure S1. Eosinophil shape change induced by PGD2, DK-PGD2, ∆12-PGD2, 15-deoxy-∆12,14-PGD2, PGJ2, 9a,11b-PGF2, ∆12-PGJ2 and 15-deoxy-∆12,14-PGJ2. Granulocytes were isolated from whole blood of asthmatic patients and were incubated with increasing concentrations of metabolites, n=3, 9α,11β-PGF2: n=2. The mean fluorescence values of the forward scatter were determined by flow cytometry and the percentage of shape change above basal was calculated. Values are given as mean ± SD. Figure S2. Concentration of IL-5 cytokine secretion of ILC2s in the presence of ascending concentrations of PGD2 and selected metabolites, n=3. Values are given for three different subjects (circle, square and triangle). Figure S3. Concentration of IL-13 cytokine secretion of ILC2s in the presence of ascending concentrations of PGD2 and selected metabolites, n=3. Values are given for three different subjects (circle, square and triangle). [file 12931_2021_1852_MOESM1_ESM.docx]

SUPPLEMENT

**Supplementary table 1.** Detailed information on flow cytometric antibodies used for cell sorting of ILC2s.

| Epitope | Dye | Clone | Company | Cat# | Concentration [µg/mL] * |
| --- | --- | --- | --- | --- | --- |
| CD4 | PerCP-Cy5.5 | A161A1 | BioLegend | 357414 | 0.25 |
| CD8 | PerCP-Cy5.5 | HIT8a | BioLegend | 300924 | 0.2 |
| CD14 | PerCP-Cy5.5 | HCD14 | BioLegend | 325622 | 0.6 |
| CD16 | PerCP-Cy5.5 | B73.1 | BioLegend | 360712 | 0.5 |
| CD19 | PerCP-Cy5.5 | HIB19 | BioLegend | 302230 | 0.3 |
| CD34 | PerCP-Cy5.5 | 581 | BioLegend | 343522 | 0.5 |
| CD123 | PerCP-Cy5.5 | 6H6 | BioLegend | 306016 | 2.5 µl** |
| FceRIa | PerCP-Cy5.5 | AER-37 | BioLegend | 334622 | 0.25 |
| CD3 | BV510 | OKT3 | BioLegend | 317332 | 0.06 |
| CD294 | PE | BM16 | Miltenyi Biotech | 130-113-600 | 2.5 µl** |
| CD45 | AlexaFluor 700 | HI30 | BioLegend | 304024 | 0.75 |
| CD56 | FITC | HCD56 | BioLegend | 318304 | 0.2 |
| CD11b | FITC | Bear1 | Beckman Coulter | IM0530 | 2.5 µl** |
| CD127 | BV421 | A019D5 | BioLegend | 351310 | 0.25 |

* per 1x10^6 cells

** antibody concentrations were not provided by supplier, antibody volume/ 1x10^6 cells is indicated

**Supplementary Figure 1**. Eosinophil shape change induced by PGD_2_, DK-PGD_2_, ∆^12^-PGD_2_, 15-deoxy-∆^12,14^-PGD_2_, PGJ_2_, 9a,11b-PGF_2_, ∆^12^-PGJ_2_ and 15-deoxy-∆^12,14^-PGJ_2_. Granulocytes were isolated from whole blood of asthmatic patients and were incubated with increasing concentrations of metabolites, n=3, 9α,11β-PGF_2_: n=2. The mean fluorescence values of the forward scatter were determined by flow cytometry and the percentage of shape change above basal was calculated. Values are given as mean ± SD.

**Supplementary Figure 2.** Concentration of IL-5 cytokine secretion of ILC2s in the presence of ascending concentrations of PGD2 and selected metabolites, n=3. Values are given for three different subjects (circle, square and triangle).

**Supplementary Figure 3.** Concentration of IL-13 cytokine secretion of ILC2s in the presence of ascending concentrations of PGD2 and selected metabolites, n=3. Values are given for three different subjects (circle, square and triangle).

**Supplementary table 2.** Calculated agonist EC_70_ values for PGD_2_ and seven selected PGD_2_ metabolites.

| Metabolite | Eosinophil shape change | ILC2 migration | ILC2 cytokine secretion |
| --- | --- | --- | --- |
| PGD_2_ | 0.9 | 27.7 | 348.2 |
| DK-​PGD_2_ | 3.9 | 22.6 | 468.3 |
| PGJ_2_ | 5.3 | 124.9 | 677.0 |
| Δ^12^-​PGJ_2_ | 6.8 | 188.6 | 759.0 |
| Δ^12^-​PGD_2_ | 1.9 | 34.7 | 364.2 |
| 15-​Deoxy-​Δ^12,​14^-​PGJ_2_ | 16.4 | 77.7 | 520.6 |
| 15-​Deoxy-​Δ^12,​14^-​PGD_2_ | 2.0 | 44.9 | 446.0 |
| 9α,11β-PGF_2_ | 1000 | 12905.5 | 1263.4 |

**Supplementary table 3.** Characteristics of study subjects. All subjects had a history of allergic asthma since at least 12 months. BMI had to be between 19 to 32 kg/ m^2^.

| Characteristics | Asthmatics [mean ± SD] |
| --- | --- |
| n | 15 |
| Gender m/f | 8/7 |
| Age | 34.5 ± 10.6 |
| Smoking status | 14 Non-smoker, 1 smoker |
| Asthma step 0/1/2/3 | 1/8/1/5 |
